# Surgeons’ personal cloth scrub caps: harmless perk or implicit infection prevention risk?

**DOI:** 10.1186/s13037-025-00465-9

**Published:** 2026-01-29

**Authors:** Jenna Hughes, Emily M. Pilc, Clifton Bridges, Hans Robert Tuten

**Affiliations:** 1 Bon Secours Tuckahoe Orthopedics, Richmond, VA USA; 2 Bon Secours Department of Pathology/Microbiology, St. Mary’s Hospital Richmond, Richmond, VA USA

**Keywords:** Surgical site infections, Scrub caps, Sterile field

## Abstract

**Background:**

Many hospitals are implementing requirements for disposable-only scrub caps in the operating room as an infection prevention measure. Current literature has not identified a significant difference in the infection risk between cloth and disposable scrub caps. This study seeks to address this issue at the cap level by determining the difference in bacterial colonization between cloth and paper scrub caps. With consideration for potential contamination associated with environments beyond the operating room, it was predicted that personal cloth scrub caps would have a higher infectious load than disposable caps.

**Methods:**

This study was designed as a prospective cohort study at a non-profit, 391 bed medical center in the United States. The sample included scrub caps from 107 medical personnel within the sterile field, including surgeons, physician assistants, nurse practitioners, surgical assistants, and scrub nurses. Medical personnel wore the cap style and material of their choice. The swab collections were cultured for 3 days on blood agar plates and were evaluated for bacterial colony growth and were quantified on the following scale: 0 – no bacterial growth, 1 – few colonies, 2 – several colonies, but less than 50% coverage, 3 – significant colonization at over 50% coverage, and 4 – full or nearly full colonization. The data was then calculated for statistical significance.

**Results:**

A total of 107 samples were obtained, including 58 from cloth scrub caps and 49 from paper scrub caps. On disposable paper caps, the mean number of colony forming units was 1.06 (SD = 3.60), and the mean growth rank was 0.20 (SD = 0.61). On reusable cloth caps, the mean number of colony forming units was 5.16 (SD = 11.78), and the mean growth rank was 0.93 (SD = 0.99). Using a Mann-Whitney U test to evaluate the effect of cap type on colony forming units and growth rank, reusable cloth caps were found to be associated with increased colony forming units (*p* < 0.001) and increased growth rank (*p* < 0.001).

**Conclusion:**

Reusable cloth scrub caps were found to have more colony forming units and higher growth ranks than disposable paper scrub caps. Attention should be given to the proper sterilization of cloth scrub caps in order to decrease the infectious load on items in the operating room.

## Introduction

The risk of surgical site infections poses a major threat to optimal post-operative patient recovery [1]. In first-world countries, 7% of surgical patients in the inpatient setting experienced surgical site infections, with a cost of 10 billion dollars annually in the United States [1]. Of adult patients in the United States undergoing total joint arthroplasty and pediatric patients undergoing spinal fusion surgeries, 3.7% and 3.6% − 10.3% of patients suffered SSIs respectively [1, 2]. Prioritizing the integrity of the sterile field is essential in reducing surgical site infections, including attention to potential pathogens located on the surgeons themselves and their clothing [3, 4]. Scrub caps have been deemed necessary in the surgical setting to reduce potentially infectious shedding through hair and other bodily materials from medical personnel leaning over the surgical site [4, 5]. Recent mandates of some institutions requiring surgical staff to use disposable scrub caps instead of cloth scrub caps have caused some discontent between medical personnel and their hospital administration nationwide [6]. Common complaints include an unnecessary limit on autonomy, with much of this concern stemming from a lack of significant evidence that disposable paper caps do in fact reduce surgical site infections (SSIs) [4].

Previous studies on the topic of surgical attire have revealed no significant difference in bacterial loads or patient infection rates between disposable and cloth garments [6–10]. However, several studies compared samples of new, paper garments with freshly laundered cloth garments, which fails to account for the contamination risks associated with reusable garments being worn outside of the operating room [4]. Paper scrub caps are designed to be disposed of after a single-use, and are easily replaced by an unused cap, while cloth scrub caps require repeated laundering between uses [6]. It is unclear if surgical administrations have policies regarding the cleanliness of cloth scrub caps that are enforceable and effective [10].

This study seeks to determine the bacterial load, measured in bacterial colonization from scrub cap cultures, while accounting for the influence of improper scrub cap hygiene. Assessing scrub caps immediately prior to the start of surgical procedures allowed for a sample including realistic laundering status, representative of the actual infection risks that patients face during surgery. With consideration for potential contamination associated with environments beyond the operating room, it was predicted that personal cloth scrub caps would have a higher infectious load than disposable caps.

## Methods

This was a prospective, comparative cohort study approved by the Bon Secours Mercy Health Institutional Review Board. Inclusion criteria were medical personnel at Bon Secours St. Mary’s Hospital and St. Mary’s Ambulatory Surgery Center in Richmond, VA within the operating room sterile field and in close proximity to the patient and surgical incision: surgeons, physician assistants, nurse practitioners, surgical assistants, and scrub nurses. Medical personnel could use their preference of disposable paper caps or cloth caps. Cap style, including Bouffant and Skull Cap style scrub caps, was not selected for, and medical personnel could use their preference of cap style. Participation in this study was voluntary and anonymous; participants could withdraw at any time. Cloth scrub caps were sampled from medical personnel entering the operating room prior to any previous surgeries, and disposable scrub caps were sampled immediately out of the cap storage unit and prior to any previous use.

**Fig. 1:**
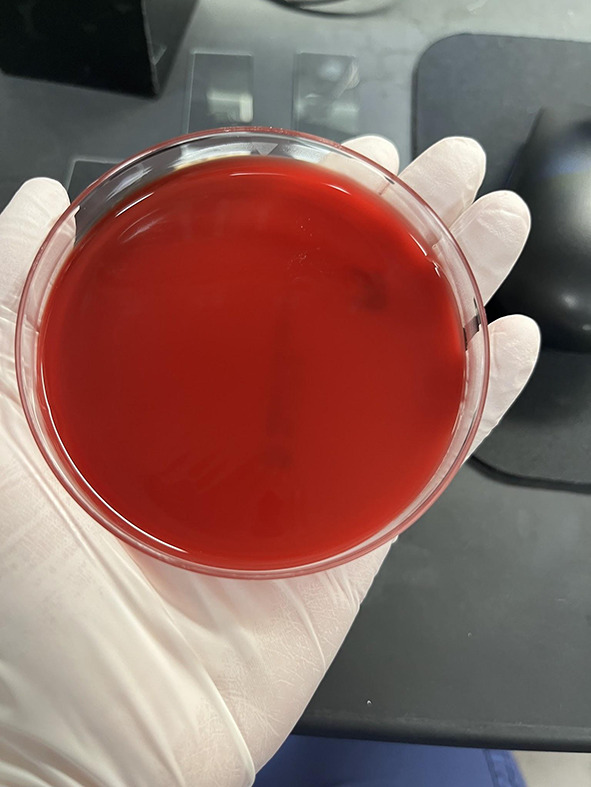
Growth rank 0

**Fig. 2:**
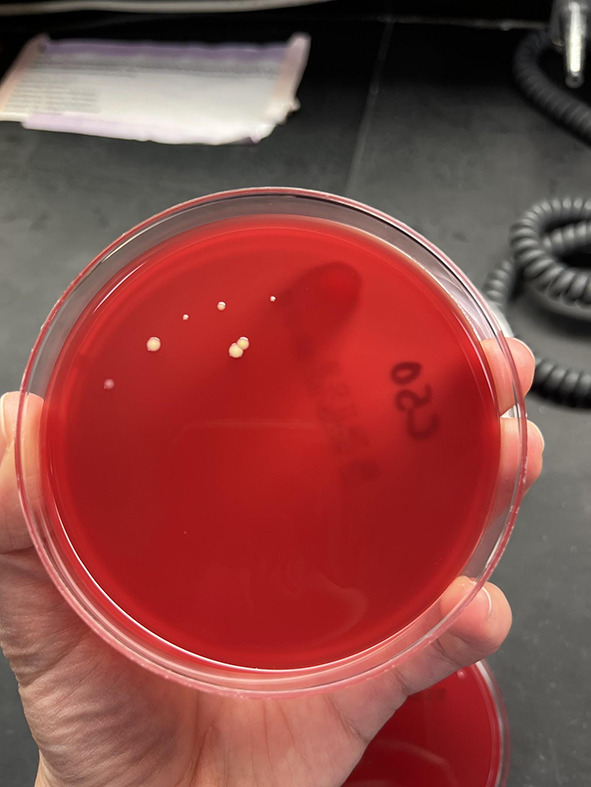
Growth rank 1

**Fig. 3:**
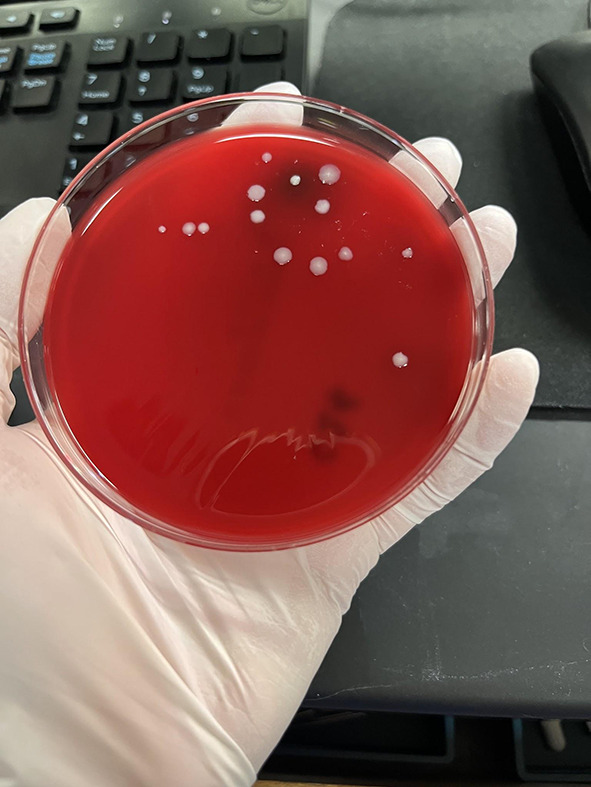
Growth rank 2

**Fig. 4:**
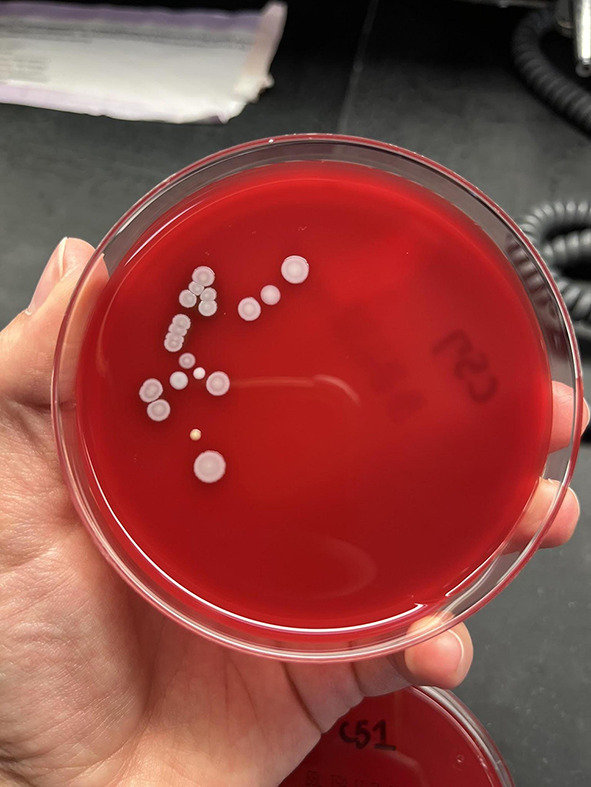
Growth rank 3

Samples were obtained using BBL CultureSwab Plus Sterile culture swabs. Each swab was transferred to one blood agar plate and was allowed to culture for 3 days in the incubator at 37 °C and 7% CO_2_ conditions. After the 3 day period, the agar plates were evaluated for the number of colony forming units and were quantified on the following growth rank scale: 0 – no bacterial growth (Fig. 1), 1 – few colonies of bacteria (Fig. 2), 2 – several bacterial colonies, but less than 50% coverage of the streaked portion of the plate (Fig. 3), 3 – significant bacterial colonization at more than 50% coverage of the streaked portion of the plate (Fig. 4), and 4 – full or nearly full colonization of the streaked portion of the plate. Additionally, the bacterial colonies were identified via visual inspection by lab personnel and through coagulase and gram staining tests in instances where visual inspection was not definitive.

Power analysis conducted through G*Power (HHU) indicated that 106 samples would be sufficient to detect a statistically significant effect (alpha = 0.05) of scrub cap type on quantified bacterial growth rank with 80% probability [power (1-β): 0.8033] The data was calculated for statistical significance using a Mann-Whitney U test.

## Results

A total of 107 scrub caps were sampled, surpassing the 106 samples sufficient to detect a significant effect calculated by the power analysis. The 107 samples included 58 reusable cloth scrub caps and 49 disposable paper scrub caps. Both Bouffant and Skull Cap style scrub caps were present in the cloth and the paper cap groups. On paper caps, the mean number of colony forming units was 1.06 (SD = 3.60), and the mean growth rank was 0.20 (SD = 0.61) (Table 1). On cloth caps, the mean number of colony forming units was 5.16 (SD = 11.78), and the mean growth rank was 0.93 (SD = 0.99) (Table 1). Using a Mann-Whitney U test to evaluate the effect of cap type on colony forming units and growth rank, reusable cloth caps were found to be associated with increased colony forming units (*p* < 0.001) and increased growth rank (*p* < 0.001) compared to disposable paper caps. Independent from the bacterial colony forming units and growth ranking data, the bacteria cultured from the scrub caps was identified as, from most to least common: coagulase-negative Staphylococcus (38 strands), Diphtheroids (7 strands), Micrococcus (3 strands), and single colonies of gram positive branching rods, alpha hemolytic Streptococcus, and *Staphylococcus aureus*.

**Table 1:** Raw data

	Paper Scrub Caps		Cloth Scrub Caps	
Specimen Number	Colony Forming Units	Growth Rank	Colony Forming Units	Growth Rank
1	0	0	0	0
2	4	1	5	1
3	0	0	1	1
4	3	1	0	0
5	13	2	50	4
6	0	0	4	1
7	0	0	0	0
8	15	2	8	2
9	0	0	6	1
10	0	0	3	1
11	0	0	63	3
12	0	0	0	0
13	0	0	10	2
14	0	0	14	2
15	0	0	2	1
16	0	0	0	0
17	0	0	1	1
18	0	0	0	0
19	0	0	38	4
20	0	0	1	1
21	0	0	0	0
22	0	0	1	1
23	0	0	1	1
24	0	0	17	2
25	0	0	3	1
26	0	0	0	0
27	0	0	1	1
28	0	0	0	0
29	0	0	1	1
30	1	1	0	0
31	0	0	2	1
32	0	0	1	1
33	0	0	2	1
34	0	0	0	0
35	0	0	6	1
36	16	3	0	0
37	0	0	1	1
38	0	0	1	1
39	0	0	0	0
40	0	0	1	1
41	0	0	0	0
42	0	0	2	1
43	0	0	1	1
44	0	0	1	1
45	0	0	3	1
46	0	0	1	1
47	0	0	0	0
48	0	0	0	0
49	0	0	16	3
50			7	1
51			2	1
52			0	0
53			0	0
54			0	0
55			0	0
56			0	0
57			20	3
58			2	2
**Mean**	1.06	0.20	5.16	0.93
**Median**	0	0	1	1
**Std Dev**	3.60	0.61	11.78	0.99
**Skew**	3.61	3.28	3.58	1.38
**Min**	0	0	0	0
**Max**	16	3	63	4

## Discussion

The data showed that reusable cloth caps grew more and larger bacterial colonies compared to paper caps, supporting the hypothesis that cloth scrub caps carried larger bacterial loads than paper caps. Surgeons’ hair is a known vehicle of transportation for infectious bacteria, particularly *Staphylococcus aureus* [2, 3]. While not the focus of the data for this study, bacterial cultures were identified for implications beyond the main data set. An overwhelming majority of the bacterial cultures in this study were identified as coagulase-negative Staphylococci. Only a single colony of *Staphylococcus aureus* was identified. Future studies are recommended to investigate the clinical significance of coagulase-negative Staphylococcus on SSIs and the low prevalence of *Staphylococcus aureus* on surgical headwear in this study, contrary to what previous studies have suggested. Based on this information, medical personnel should be mindful of these differences in bacterial loads when choosing their surgical headwear.

Given that cloth, reusable scrub caps were found to contain higher bacterial loads, this then begs the question of how to properly clean reusable garments and equipment used in the operating room. In a study investigating the use of stuffed animals for emotional comfort in pediatric surgery, all stuffed animals in the sample were found to have positive bacterial cultures [11]. However, a large majority of the stuffed animals were deemed sterile after a single wash and dry cycle in a conventional home machine one night prior to surgery, and sterility was maintained up to the procedure by sealing the stuffed animal in a bag [11]. Applying this to the question of reusable personal scrub caps, the infectious load of cloth scrub caps may be better managed by proper laundering, drying, and transportation prior to the procedure.

When comparing the infection risk of cloth versus disposable scrub caps at a material level, consideration must be given to the porosity of the cap types, as increased porosity suggests an increased potential to harbor particles and micro-organisms [4]. Previous studies show that disposable caps, specifically in the Bouffant style, have significantly larger maximum pore size in comparison to cloth caps, specifically in the Skull Cap style [4]. Furthermore, in a study by Markel et al., disposable Bouffant caps produced more colony forming units of bacteria in comparison to freshly laundered cloth Skull Caps when swabbed and cultured [4]. No significant difference in SSIs was found between patients operated on by surgeons using disposable Skull Caps and by surgeons using disposable Bouffant style caps [10]. This suggests that regardless of cap style, the material of disposable caps may harbor more infectious organisms than freshly laundered personal cloth scrub caps. These findings contradict the results of our study, as disposable caps were found to have significantly lower colony forming units and growth ranks than cloth caps. Of note, our study did not select for the laundering status of cloth caps, which suggests that cap material is not the driving factor in the caps’ bacterial loads.

Infectious material on scrub caps is not the only factor regarding scrub cap selection and patient safety. Other studies have evaluated the use of cloth scrub caps personalized with medical personnel’s names, roles, and other identifiers in an effort to improve team communication in the operating room [6]. Nyima et al. summarized several studies that found embroidered scrub caps improved operating procedure efficiency by reducing communication delays [6]. Bungert et al. assessed this aspect of surgical scrub caps via disposable scrub caps with name tag stickers attached and found that name tagged scrub caps improved overall communication and delegation of tasks [12]. However, concern was raised that scrub cap name tags have the potential to become a surgical field contaminant themselves, in the event that the name tag fails to adhere to the scrub cap for the full extent of the procedure [12]. In regards to this study, neither disposable nor cloth scrub caps possessed any form of surgical personnel identification.

This study is not without limitations. One limitation is that the sample size and population diversity were limited due to sample selection from a single hospital and ambulatory surgery center. However, our sample size surpassed the number calculated in the power analysis necessary to detect a difference between the two groups. An additional limitation is that cultures were grown for three days, so contamination with yeast could not be evaluated. While this study did not evaluate clinical outcomes of scrub cap contamination, given that the most common SSI pathogens are bacterial as opposed to yeast, this study focused on bacterial infectious loads [5, 13]. Another limitation is that the disposable paper caps were swabbed directly out of their containers before the first case of the day. In reality, surgical personnel may wear the same cap throughout the day, exposing it to potential contamination prior to later cases. Had the caps been swabbed later in the day, the results may have differed. However, by swabbing the caps in their pristine, unused state, we were able to establish a baseline level of contamination directly attributable to the caps themselves, independent of environmental or personnel-related factors. This approach strengthens the internal validity of our comparison by isolating the variable of cap material.

## Conclusion

Surgical site infections are both taxing on patients’ health and well-being, as well as very expensive for hospitals to treat. In an effort to both protect patients and improve hospital financial efficiency, more attention should be given to potential sources of infectious bacteria. This study approached this issue with a focus on surgical headwear and found that reusable, cloth scrub caps carried a greater bacterial load, both in colony quantity and size, than disposable paper scrub caps, and thus have a greater potential for surgical site infection when laundering status is not controlled. From this data, more attention should be given to the proper cleansing of reusable scrub caps, and further studies could be conducted to assess the surgical site infection rates of disposable scrub caps compared to reusable scrub caps without selecting for freshly laundered status.

## Data Availability

All data gathered or analysed during this study are included in this published article.
